# Prevalence and Persistence of Cost-Related Medication Nonadherence Among Medicare Beneficiaries at High Risk of Hospitalization

**DOI:** 10.1001/jamanetworkopen.2021.0498

**Published:** 2021-03-03

**Authors:** Jorge L. De Avila, David O. Meltzer, James X. Zhang

**Affiliations:** 1 Pritzker School of Medicine, The University of Chicago, Chicago, Illinois; 2 Department of Medicine, The University of Chicago, Chicago, Illinois; 3 Harris School of Public Policy, The University of Chicago, Chicago, Illinois; 4 Department of Economics, The University of Chicago, Chicago, Illinois

## Abstract

**Importance:**

The unaffordability of drugs has been a persistent and elusive challenge in the US health care system. Little is known about the prevalence and persistence of cost-related medication nonadherence (CRN) in a population with high-cost, high-need resource utilization.

**Objective:**

To evaluate the prevalence and persistence of CRN among Medicare beneficiaries at high risk of hospitalization as well as the characteristics associated with CRN in this population.

**Design, Setting, and Participants:**

This cohort study used survey data from Medicare patients at high risk of hospitalization and with a life expectancy greater than 12 months at an urban academic medical center from November 6, 2012, to January 30, 2018. Patients were followed up for 12 months at 3-month intervals from baseline, for a total of 5 surveys. Data were analyzed from September 1, 2020, to January 5, 2021.

**Main Outcomes and Measures:**

Self-reported CRN, using a metric of persistence and transiency. Based on the results of the 5 surveys, CRN was categorized as persistent (3 or more surveys), intermittent (2), transient (1), and any (1 or more). Multiple logistic regression analyses were used to evaluate factors associated with persistent and transient CRN.

**Results:**

Of the 1655 Medicare beneficiaries followed up during the 15-month study period, 1036 (62.6%) were women and 1452 (87.7%) were Black or African American; 769 (46.5%) were younger than 65 years, and 886 (53.5%) were 65 years or older (mean [SD] age, 62.4 [15.9] years). A total of 374 patients (22.6%) reported CRN at baseline, 810 (48.9%) reported any CRN, and 230 (13.9%) reported persistent CRN (148 [19.2%] of those younger than 65 years and 82 [9.3%] of those 65 years or older). The 230 patients who had persistent CRN accounted for 28% of those who reported CRN at least once during the 15-month study period. Younger age (eg, <50 years vs 75 years: adjusted odds ratio [AOR], 3.07; 95% CI, 1.61-5.86; *P* = .001), worse self-reported health (AOR, 1.59; 95% CI, 1.10-2.31; *P* = .01), and depression (AOR, 1.58; 95% CI, 1.11-2.24; *P* = .01) were associated with greater likelihood of persistent CRN. The population-adjusted prevalence of CRN was 53.6% (887 patients).

**Conclusions and Relevance:**

The findings suggest that CRN is prevalent, moderately persistent, and variable in the Medicare population at high risk of hospitalization despite coverage by insurance. Longitudinal follow-up and refined predictive modeling of CRN appear to be needed to identify and target more precisely those with persistent CRN and to develop effective interventions.

## Key Points

**Question:**

What are the prevalence and persistence of cost-related medication nonadherence (CRN) among Medicare beneficiaries at high risk of hospitalization, a population with high-cost, high-need resource utilization, and what is the longitudinal pattern of CRN over time?

**Findings:**

In this cohort study of 1655 Medicare beneficiaries, the population-adjusted prevalence of CRN was 53.6%, and 28.4% of those who reported CRN at least once had persistent CRN during the 15-month study period. Younger age, worse self-reported health, and depression were associated with greater likelihood of persistent CRN.

**Meaning:**

The findings suggest that CRN is prevalent, moderately persistent, and variable in the Medicare population at high risk of hospitalization despite coverage by insurance.

## Teaser

This cohort study evaluates the prevalence and persistence of cost-related medication nonadherence (CRN) among Medicare beneficiaries at high risk of hospitalization as well as the characteristics associated with CRN in this population.

## Introduction

High pharmaceutical drug prices have been a persistent and elusive challenge for the US health care system.^1^ One in 4 adults in the US has a difficult time affording their medications,^2^ and among Medicare beneficiaries in 2006 11.5% reported medication nonadherence owing to financial barriers.^3^ Numerous behavioral, social, economic, medical, and policy-related factors are associated with medication nonadherence,^4,5,6,7,8,9,10,11,12^ and medication nonadherence is associated with increased hospitalization rates and emergency department visits, higher mortality rates, worse patient outcomes, and increased downstream costs, all of which impose avoidable and substantial health care costs on society.^13,14,15,16,17,18^ With use of cross-sectional data sets over time, researchers have found that although Medicare Part D has provided outpatient prescription drug coverage for Medicare beneficiaries since its implementation in 2006, the prevalence of cost-related medication nonadherence (CRN) has actually increased among the sickest Medicare beneficiaries, including older patients with complex medical needs and people with disabilities.^19,20,21,22^ Although these cross-sectional estimates showed the aggregate trend in CRN for the populations studied, because the individuals were not longitudinally linked, the level of CRN behaviors, such as persistence and transiency, are unclear. Without knowledge of the key characteristics associated with CRN behaviors, targeting individuals who report CRN occasionally for intervention may be economically inefficient and fail to focus on those who are persistently unable to pay for medications. In addition, the lack of longitudinal follow-up may lead to underestimation of the prevalence rate of CRN and to distorted risk profiles for patients at high risk of CRN.

It is estimated that in the US, patients with high-need, high-cost resource utilization (approximately 5% of the population) disproportionately account for 50% of all healthcare spending,^23^ and the Medicare population at high risk of hospitalization well represents this group because many are older adults, have multiple chronic conditions, or experience extreme functional limitations. Although this group has been the topic of many policy discussions, little is known about their behaviors with regard to CRN because physicians and patients infrequently discuss CRN and, when they do, both parties are often frustrated by the lack of a clear solution.^24^ In addition, most of the literature^3,10,12^ on CRN anchors its measure of prevalence on a 1-time cross-sectional survey, implicitly assuming that a 1-time measure would be persistent or stable during the recall period, such as 1 year. However, if CRN is not static or binary, a 1-time cross-sectional measure may focus an intervention on individuals with transient CRN and fail to target those who have persistent difficulty paying for medications and therefore require structured financial assistance. This is a particularly important distinction for practice and policy in the Medicare population because all patients have public insurance, with some having both Medicare and Medicaid to help cover their coinsurance and premium.^25^ A high prevalence of CRN in this population would suggest inadequacy of insurance coverage, poor benefit design for drugs, and/or high price sensitivity by the patients, all of which would require policy action to improve insurance coverage and reduce downstream costs associated with CRN behaviors.

Therefore, we evaluated the prevalence, persistency, and transiency of CRN over time in a sample of Medicare beneficiaries at high risk of hospitalization. Understanding the CRN behaviors in this population over time is important because nonadherence to medication may be associated with worse health outcomes and higher downstream costs compared with those in other populations. We also assessed potential factors associated with persistent CRN by analyzing the patients’ sociodemographic and health characteristics and how these factors may be associated with the protective effect of Medicare coverage.

## Methods

This cohort study used survey data from Medicare patients at high risk of hospitalization who previously enrolled in a study of the Comprehensive Care Physician model at an urban academic medical center.^26^ The enrollment criterion was hospitalization at least once in the past year or emergency department care at the time of enrollment. The study was approved by The University of Chicago institutional review board, with written informed consent provided by participants. This study followed the Strengthening the Reporting of Observational Studies in Epidemiology (STROBE) reporting guideline.

Our internal analysis indicated that the annual health costs for patients fulfilling the enrollment criterion were 300% to 400% of the mean annual health cost for Medicare beneficiaries during the follow-up period. The CRN rate in this population at baseline was likely higher than that in the general population because the patients fulfilling the enrollment criterion likely had higher resource utilization and severity of illness. This criterion allowed us to study the persistency of CRN in a population at increased risk for CRN. The enrollment and study period was from November 6, 2012, to January 30, 2018, during which the US economy steadily recovered from the Great Recession in 2008; thus, data could be analyzed for patient CRN behaviors without major economic volatility. Although the study on the Comprehensive Care Physician model was a randomized clinical trial, for the present study, we pooled data from the study and control arms because our interest was primarily tracking the pattern of CRN rather than the effect of the model on CRN over time. We controlled for the study arm in the multiple regression analysis to reflect the population-mean CRN adjusted for other confounding factors. We also conducted a stratified analysis of the trajectory of CRN rates in the 2 study arms over time to ascertain whether the CRN rates were similar during this study period.

Our study included 5 surveys of CRN: a baseline survey of CRN in the 3 months before study enrollment and 4 follow-up surveys at 3-month intervals in the subsequent 12 months. Thus, the total study period covered by the CRN questionnaires was 15 months. To fully capture the CRN trajectory for the complete study sample, we excluded 54 patients without baseline data, 7 who eventually withdrew from the study, 61 who did not complete all 4 follow-up surveys, and 223 who died during the 12-month period, resulting in a total of 1655 participants in this study. Patients who died within 12 months after enrollment had a short life expectancy, and we did not have enough observation points to construct a comparable measure of prevalence and persistence over time. In addition, resource utilization among Medicare patients in their last year of life accounts for one-quarter of Medicare spending.^27,28,29^ Such heterogeneity in resource utilization patterns (and thus costs to patients) and its association with patients’ behaviors requires a separate investigation of CRN to avoid confounding of research.

Patients who did not answer CRN questionnaires intermittently during the 12-month follow-up period after the baseline survey were considered as not reporting CRN for that observation. The overall response rate for the follow-up surveys was 93%. Among the respondents, 2.9% of responses were “don’t know” or “refusal” to 1 or more of the 4 CRN questions on the survey.

The CRN was self-reported based on 4 questions that were adopted from the Medicare Current Beneficiary Survey,^3^ with a recall timeframe set at 3 months instead of 1 year. The survey included the following question: “during the past 3 months, have you ever done the following due to cost: (1) not fill or refill a prescription, (2) delay filling a prescription, (3) skip doses, or (4) take smaller doses to make medication last longer.” The CRN was categorized as 1 if the patients reported any of these 4 options and 0 if they did not report any option. Conceptually, the 3-month timeframe may have reduced the recall bias and provided more detailed information about CRN behavior than the 12-month recall questionnaires. A metric was developed to measure the CRN longitudinally. *Any CRN* was defined as reporting CRN on at least 1 survey during the study period; *baseline CRN*, on the enrollment survey; *transient CRN*, on 1 survey; *intermittent CRN*, on 2 surveys; and *persistent CRN*, on 3 or more surveys. To our knowledge, no studies have attempted to define *persistence* in CRN longitudinally; defining *persistent CRN* as reporting CRN during more than half of the study period (ie, 3 of 5 surveys) indicated that the patient was having CRN more often than not having CRN. Such measures of CRN may provide cumulative frequency, transiency, and persistence over time.

Demographic and health characteristics collected at baseline were included to examine variations in CRN between subgroups in the study population and to adjust for confounders to evaluate the population-mean factors associated with CRN. These factors included age, gender, race/ethnicity, educational level, health literacy,^30^ income, insurance, self-reported health, health conditions (cancer; cardiovascular disease, including angina, congestive heart failure, and coronary artery disease; depression; kidney disease; and diabetes), hospitalizations in the past 12 months, and study group (standard of care vs intervention). These factors have been reported to be associated with CRN in cross-sectional studies,^4,5,6,7,8,9,10,11,12^ but how they are associated with CRN longitudinally in a population with high-cost, high-resource utilization is unknown. Of note, although the surveys asked for reports of sex, because such reports encompass a cultural indicator of a person's personal social and cultural identity rather than the biological characteristics of males and females, we use gender instead of sex in the context of CRN behaviors; such behaviors are beyond the biological difference owing to differential cultural roles by men and women. There has been robust discussion in the literature^31,32,33,34^ regarding sex differences in the use of health care resources across the life span and the role of gender in contributing to such differences when socioeconomic contexts are incorporated in medical care. Although the literature on gender difference in CRN has increased,^34^ to our knowledge, no study has specifically focused on the association of gender with persistence of CRN.

Demographic and health characteristics and CRN were stratified by age (<65 years and ≥65 years) because patients younger than 65 years who were covered by Medicare were mostly those with disability or end-stage kidney disease and had disease profiles and health care needs that were different from those 65 years or older.^12^ Research has shown that this age cutoff may be associated with a difference in CRN behaviors.^12^

### Statistical Analysis

Data analysis was performed from September 1, 2020, to January 5, 2021. We conducted multiple logistic regression analyses to evaluate the potential factors associated with persistent and transient CRN. We conducted a bivariate analysis on the association of gender with transient, intermittent, persistent, and no CRN using χ^2^ tests. A multiple regression analysis of the association of gender with the persistence and transiency of CRN was performed, adjusting for other sociodemographic and health variables.

To obtain population-adjusted estimates, we conducted multiple logistic regression analyses for overall CRN, controlling for demographic and health characteristics in the full sample. *P* = .05 was considered statistically significant for 2-sided tests. The analyses were conducted using Stata, version 15.0 (StataCorp LLC).

## Results

Of the 1655 Medicare beneficiaries followed up during the 15-month study period, 1036 (62.6%) were women and 1452 (87.7%) were Black or African American; 769 (46.5%) were younger than 65 years, and 886 (53.5%) were 65 years or older (mean [SD] age, 62.4 [15.9] years). Table 1 shows the patient demographic and health characteristics and CRN stratified by age group for the 4 types of CRN. Overall, 490 patients (63.7%) younger than 65 years were dually eligible for Medicare and Medicaid compared with 305 patients (34.4%) 65 years or older (*P* < .001). Gender, race, educational attainment, health literacy, income, self-reported health, certain health conditions (cardiovascular disease, depression, diabetes, and kidney disease), and number of hospitalizations in the previous year were significantly different between patients younger than 65 years and those 65 years or older. Among those who reported CRN, 148 (33.4%) reported persistent CRN in the group younger than 65 years and 82 (22.3%) reported persistent CRN in the group 65 years or older. The population-adjusted CRN prevalence was 53.6% (887 patients) after controlling for all sociodemographic and health variables (Table 1), which is 366% higher than the reported CRN prevalence among the general Medicare population in 2006.

**Table 1.** Cost-Related Medication Nonadherence Outcomes by Demographic and Health Characteristics Stratified by Age

Characteristic	Patients, No. (%)^a^								*P* value^b^
	CRN outcome among patients aged <65 y (n = 769)				CRN outcome among patients aged ≥65 y (n = 886)				
	None	Transient	Intermittent	Persistent	None	Transient	Intermittent	Persistent	
Total patients	326 (42.4)	185 (24.1)	110 (14.3)	148 (19.2)	519 (58.6)	202 (22.8)	83 (9.4)	82 (9.3)	NA
Age, y									
<50	118 (47.0)	54 (21.5)	28 (11.2)	51 (20.3)	NA	NA	NA	NA	NA
50-64	208 (40.2)	131 (25.3)	82 (15.8)	97 (18.7)	NA	NA	NA	NA	
65-74	NA	NA	NA	NA	276 (54.2)	124 (24.4)	49 (9.6)	60 (11.8)	
≥75	NA	NA	NA	NA	243 (64.5)	78 (20.7)	34 (9.0)	22 (5.8)	
Gender									
Man	144 (45.1)	79 (24.8)	43 (13.5)	53 (16.6)	177 (59.0)	81 (27.0)	27 (9.0)	15 (5.0)	.001
Woman	182 (40.4)	106 (23.6)	67 (14.9)	95 (21.1)	342 (58.4)	121 (20.7)	56 (9.6)	67 (11.4)	
Race/ethnicity									
White	16 (43.2)	10 (27.0)	4 (10.8)	7 (18.9)	51 (68.9)	9 (12.2)	3 (4.1)	11 (14.9)	.03
Black or African American	290 (42.0)	166 (24.0)	102 (14.8)	133 (19.3)	433 (56.9)	184 (24.2)	76 (10.0)	68 (8.9)	
Hispanic or Latino	13 (38.2)	9 (26.5)	5 (14.7)	7 (20.6)	22 (73.3)	3 (10.0)	2 (6.7)	3 (10.0)	.30
Educational level									
<High school	66 (45.2)	45 (30.8)	20 (13.7)	15 (10.3)	129 (53.8)	57 (23.8)	28 (11.7)	26 (10.8)	<.001
High school	108 (43.6)	62 (25.0)	33 (13.3)	45 (18.2)	136 (61.5)	43 (19.5)	20 (9.1)	22 (10.0)	
Some college	100 (39.2)	57 (22.4)	36 (14.1)	62 (24.3)	136 (58.6)	53 (22.8)	23 (9.9)	20 (8.6)	
College graduate	48 (45.3)	18 (17.0)	17 (16.0)	23 (21.7)	97 (59.5)	42 (25.8)	10 (6.1)	14 (8.6)	
Limited health literacy	151 (43.6)	85 (24.6)	49 (14.2)	61 (17.6)	290 (58.8)	108 (21.9)	48 (9.7)	47 (9.5)	<.001
Income, $									
<15 000	115 (39.4)	75 (25.7)	37 (12.7)	65 (22.3)	158 (53.9)	80 (27.3)	31 (10.6)	24 (8.2)	<.001
15 000-25 000	33 (39.8)	14 (16.9)	17 (20.5)	19 (22.9)	52 (59.1)	19 (21.6)	7 (8.0)	10 (11.4)	
≥25 000	28 (48.3)	12 (20.7)	11 (19.0)	7 (12.1)	88 (62.9)	31 (22.1)	12 (8.6)	9 (6.4)	
Did not know or refused	101 (48.8)	45 (21.7)	31 (15.0)	30 (14.5)	148 (60.2)	47 (19.1)	23 (9.4)	28 (11.4)	
Missing	49 (38.0)	39 (30.2)	14 (10.9)	27 (20.9)	73 (61.3)	25 (21.0)	10 (8.4)	11 (9.3)	
Insurance									
Medicare only	100 (35.8)	65 (23.3)	50 (17.9)	64 (22.9)	329 (56.6)	132 (22.7)	63 (10.8)	57 (9.8)	<.001
Medicare and Medicaid	226 (46.1)	120 (24.5)	60 (12.2)	84 (17.1)	190 (62.3)	70 (23.0)	20 (6.6)	25 (8.2)	
Self-reported health									
Good, very good, or excellent	126 (49.2)	51 (19.9)	39 (15.2)	40 (15.6)	256 (64.0)	85 (21.3)	34 (8.5)	25 (6.3)	<.001
Fair or poor	199 (38.9)	134 (26.2)	71 (13.9)	108 (21.1)	262 (54.1)	116 (24.0)	49 (10.1)	57 (11.8)	
Health condition									
Cancer	16 (34.8)	9 (19.6)	11 (23.9)	10 (21.7)	43 (61.4)	17 (24.3)	6 (8.6)	4 (5.7)	.24
Cardiovascular disease	109 (40.5)	65 (24.2)	42 (15.6)	53 (19.7)	198 (54.9)	93 (25.8)	37 (10.3)	33 (9.1)	.03
Depression	78 (30.2)	66 (25.6)	51 (19.8)	63 (24.4)	100 (52.9)	43 (22.8)	20 (10.6)	26 (13.8)	<.001
Diabetes	109 (45.6)	61 (25.5)	27 (11.3)	42 (17.6)	173 (55.3)	72 (23.0)	36 (11.5)	32 (10.2)	.004
Kidney disease	97 (50.5)	49 (25.5)	21 (10.9)	25 (13.0)	97 (50.5)	49 (25.5)	14 (7.8)	17 (9.5)	.03
Hospitalizations, mean (SD)	1.68 (2.05)	1.98 (2.42)	1.79 (2.95)	2.52 (3.81)	1.32 (1.47)	1.46 (1.55)	1.44 (1.85)	1.48 (1.58)	<.001^c^
Study group									
Intervention	155 (41.6)	88 (23.7)	56 (15.1)	73 (19.6)	266 (57.5)	103 (22.3)	49 (10.6)	45 (9.7)	.12
Standard of care	171 (43.1)	97 (24.4)	54 (13.6)	75 (18.9)	253 (59.8)	99 (23.4)	34 (8.0)	37 (8.8)	

Abbreviations: CRN, cost-related medication nonadherence; NA, not applicable.

^a^ All percentages were calculated using row subtotals. The overall population-adjusted CRN prevalence was 53.6%.

^b^ *P* values were calculated using χ^2^ tests comparing percentage differences between groups <65 years and ≥65 years, regardless of CRN outcome.

^c^ *P* value for hospitalizations was calculated using *t* test.

**Figure 1.**
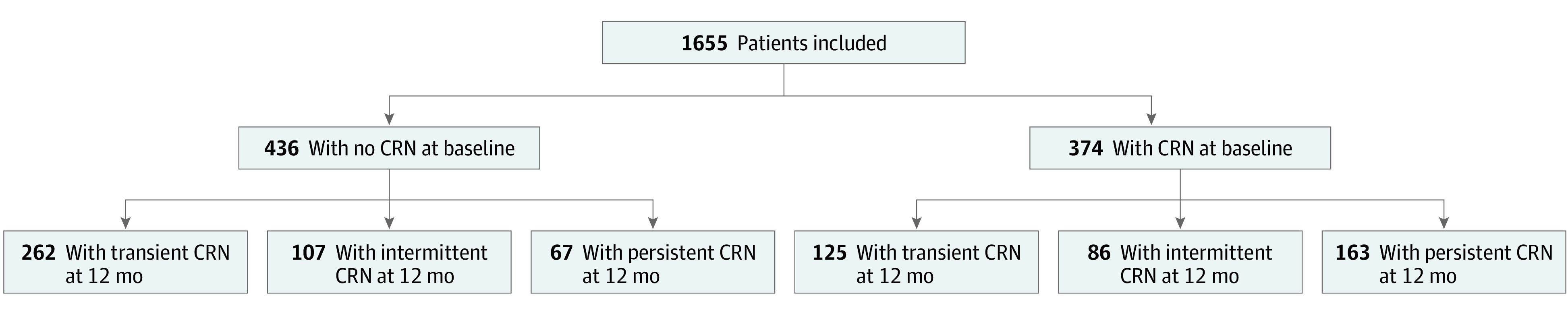
Cost-Related Medication Nonadherence (CRN) Outcomes Measured at Baseline and Throughout the Study Period

Figure 1 shows the prevalence of CRN behaviors at baseline and during the study period. Although 374 patients (22.6%) reported CRN at baseline, 810 (48.9%) had reported CRN at least once by the end of the 15-month study period. A total of 163 patients (9.8%) with baseline CRN had reported persistent CRN by the end of the study period, and 67 patients (4.0%) without baseline CRN had reported persistent CRN by the end of the study period.

**Figure 2.**
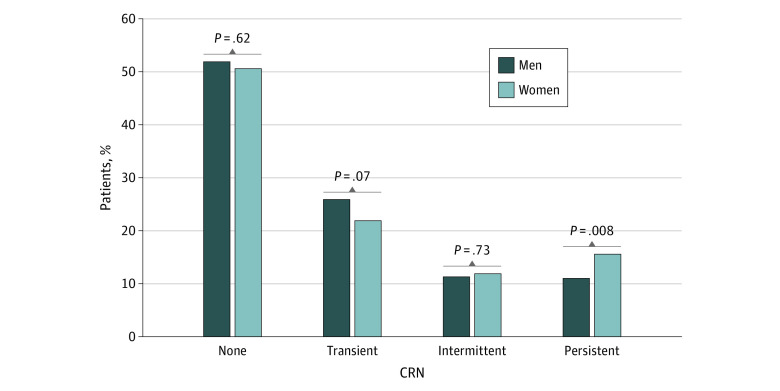
Percentage of Patients With No, Transient, Intermittent, and Persistent Cost-Related Medication Nonadherence (CRN) by Gender

Figure 2 shows of the results of the bivariate analysis of patients with no, transient, intermittent, and persistent CRN by gender. Women had lower rates of no CRN (524 [50.6%] vs 321 [51.9%]; *P* = .62) and transient CRN (227 [21.9%] vs 160 [25.9%]; *P* = .62) but higher rates of intermittent CRN (123 [11.9%] vs 70 [11.3%]; *P* = .73) and persistent CRN (162 [15.6%] vs 68 [11.0%]; *P* = .008); the difference was only significant for persistent CRN.

Table 2 gives the associations of demographic and health characteristics with CRN outcomes generated from multiple logistic regression analyses for both transient and persistent CRN. The following characteristics were associated with greater likelihood of persistent CRN: younger age (<50 years vs 75 years: adjusted odds ratio [AOR], 3.07 [95% CI, 1.61-5.86]; *P* = .001; 50-64 years vs 75 years: AOR, 3.52 [95% CI, 2.02-6.13]; *P* < .001; and 65-74 years vs 75 years: AOR, 2.24 [95% CI, 1.27-3.94]; *P* = .005), worse self-reported health (AOR, 1.59 95% CI, 1.10-2.31; *P* = .01), and depression (AOR, 1.58; 95% CI, 1.11-2.24; *P* = .01). Women had an increased risk for persistent CRN compared with men (AOR: 1.36; 95% CI, 0.95-1.95; *P* = .10), but the difference was not statistically significant. These variables were not significantly associated with transient CRN.

**Table 2.** Association Between Demographic and Health Characteristics and CRN Outcomes

Characteristic	Transient CRN		Persistent CRN	
	OR (95% CI)	*P* value	OR (95% CI)	*P* value
Age, y				
≥75	1 [Reference]	NA	1 [Reference]	NA
<50	1.16 (0.71-1.88)	.55	3.07 (1.61-5.86)	.001
50-64	1.17 (0.79-1.71)	.43	3.52 (2.02-6.13)	<.001
65-74	1.25 (0.86-1.80)	.20	2.24 (1.27-3.94)	.005
Gender				
Man	1 [Reference]	NA	1 [Reference]	NA
Woman	0.81 (0.62-1.07)	.15	1.36 (0.95-1.95)	.10
Race/ethnicity				
White	1 [Reference]	NA	1 [Reference]	NA
Black or African American	1.38 (0.76-2.52)	.29	0.87 (0.44-1.70)	.68
Hispanic or Latino	0.65 (0.27-1.54)	.33	1.24 (0.48-3.16)	.66
Educational level				
Some college	1 [Reference]	NA	1 [Reference]	NA
<High school	1.22 (0.85-1.77)	.29	0.64 (0.40-1.04)	.07
High school	0.91 (0.64-1.29)	.60	0.79 (0.52-1.20)	.27
College graduate	1.05 (0.70-1.58)	.82	0.73 (0.44-1.21)	.23
Limited health literacy	1.00 (0.76-1.34)	.96	0.97 (0.68-1.37)	.86
Income, $				
≥25 000	1 [Reference]	NA	1 [Reference]	NA
<15 000	1.23 (0.80-1.87)	.35	1.79 (0.98-3.28)	.06
15 000-25 000	0.85 (0.50-1.44)	.54	1.96 (0.98-3.91)	.06
Insurance				
Medicare and Medicaid	1 [Reference]	NA	1 [Reference]	NA
Medicare only	1.09 (0.82-1.45)	.57	1.44 (1.01-2.05)	.045
Self-reported health				
Good, very good, or excellent	1 [Reference]	NA	1 [Reference]	NA
Fair or poor	1.28 (0.96-1.69)	.09	1.59 (1.10-2.31)	.01
Health condition				
Cancer	0.88 (0.52-1.47)	.61	0.94 (0.49-1.83)	.86
Cardiovascular disease	1.18 (0.90-1.55)	.24	1.10 (0.78-1.55)	.60
Depression	1.06 (0.78-1.44)	.71	1.58 (1.11-2.24)	.01
Diabetes	1.08 (0.81-1.44)	.58	0.88 (0.61-1.26)	.49
Kidney disease	0.94 (0.68-1.29)	.68	0.84 (0.56-1.27)	.41
Hospitalizations	0.97 (0.90-1.03)	.33	1.08 (1.02-1.15)	.02
Study group				
Standard of care	1 [Reference]	NA	1 [Reference]	NA
Intervention	0.92 (0.71-1.20)	.55	0.94 (0.67-1.30)	.70

Abbreviations: CRN, cost-related medication nonadherence; NA, not applicable; OR, odds ratio.

## Discussion

Cost-related medication nonadherence was widely reported by patients in this Medicare population at high risk of hospitalization despite coverage by Medicare insurance, with a prevalence rate 366% higher than that of national mean prevalence for Medicare beneficiaries (based on an annual estimate from 2006).^3^ This finding suggests that Medicare insurance coverage may not be adequate and the benefit design may need to be improved. Literature^35,36^ suggests that, over time, Medicare patients fell into the donut hole, a coverage gap for drugs in the Part D program that ended in 2020, and that this gap was associated with worse patient outcomes. For some patients in this population, the gap may have been associated with a high rate of CRN. However, most of the patients were dually eligible for Medicare and Medicaid and thus automatically qualified for extra help paying for Medicare drug coverage (Part D), co-payments, and premiums, leaving them unaffected by the donut hole gap.^37^ In addition, patients with low income who were not dually eligible could receive extra help elsewhere.^38^ Many Medicare drug insurance programs have formularies that require high out-of-pocket payments for drugs that are not preferred or are outside formularies.^39^ High out-of-pocket costs for drugs outside formularies may have been associated with CRN, and high sensitivity to drug prices among patients with low income may further contribute to such behaviors. More research investigating the interaction among out-of-pocket payments, formularies, and price sensitivity in this population is needed to better understand the dynamics in the decision-making process of these patients.

This study revealed that CRN was cumulative and increased significantly during the 15-month study period. Even with a high CRN rate at baseline, the CRN rate continued to increase and more than doubled at the end of the year of follow-up. Although many patients reported CRN over time, not all CRN was persistent, and the pattern of persistence varied by age group. For example, among those who reported CRN, 33.4% reported persistent CRN in the group younger than 65 years and 22.3% reported CRN in the group 65 years or older. This difference in CRN associated with age was found even after controlling for all other socioeconomic and health characteristics. Patients with persistent CRN should be the focus of interventions to reduce CRN because CRN may be associated with greater harm to their health. However, a cross-sectional estimate of the CRN in the current literature^3^ has not captured the strength of this association because, to our knowledge, there is no information about the intensity of CRN measured by the persistence by individual patient over time. Therefore, the approach to addressing CRN may need to be differentiated for those younger than 65 years and those 65 years or older because, in the present study, younger age was associated with greater odds of persistent CRN.

This study also suggests that an approach to addressing CRN stratified by gender may be important. From this longitudinally followed-up sample, women had significantly greater odds of persistent CRN in the bivariate analysis, but the difference with men was not significant after adjustment for socioeconomic and health variables in the multiple regression analysis. This finding may suggest that gender is a composite variable reflecting many aspects of social, economic, and health factors that are differentially associated with CRN. Research has identified gender as a factor significantly associated with CRN to a broad range of medical care, including medications, diagnostic tests, and follow-up visits.^34^ Together, these data suggest the need for a gender-conscious approach because the challenges to pay medical care experienced by men and women may be different and their internal decision-making process may also be different.

Of note, many patients who reported baseline CRN had transient CRN over the 1-year follow-up period. This finding suggests that although 1-time cross-sectional surveys of CRN may capture the prevalence of CRN at a certain time point, this measure may not be sufficient in terms of understanding the gravity of CRN in a population such as the one included in this study. The literature^40^ has shown that interventions to reduce CRN are often costly and ineffective, and the lack of focus on patients with persistent CRN may be a reason why an intervention based on a 1-time measure of CRN may fail to identify patients with persistent CRN.^3,12^

This study revealed potential factors associated with persistent CRN. In addition to age, other factors, including poor general health perception and depression, were associated with higher persistence of CRN. These findings suggest that refined predictive modeling is needed to identify patients at high risk of CRN by including multiple factors associated with CRN, including age, health perception, and depression, and possibly a broad set of other variables that were not significantly associated with CRN in this study but may be with use of a larger sample.

Our study also showed that transient CRN was more likely than persistent CRN to be a random process because many factors that were significantly associated with persistent CRN were not significantly associated with transient CRN. This finding is important because people may have a variety of motivations for nonadherence to medications. For example, our internal discussions with patients revealed that sometimes gifts to children or grandchildren are associated with CRN because the patients deplete their disposable income. Such CRN behavior has an altruistic motivation and does not necessarily require a policy action or intervention, and thus, it is important to properly identify persistent CRN because these instances of transient CRN should not be a focus of medical intervention.

### Limitations

This study has limitations. First, self-reporting is always affected by recall bias and may also be affected by a sense of shame when reporting CRN; thus, the CRN rates reported in this study may be underestimates. We aimed to reduce recall bias in this study by setting the recall time to 3 months instead of the 12-month period used in most studies.^3,12^ Second, we excluded patients who died during the 12-month study period. Medicare patients have high resource utilization during their last year of life,^27,28,29^ and their daily life and medical needs are important research topics beyond the scope of this study. Third, the patients cared for at this urban academic medical center are predominantly Black or African American; thus, the findings may not be generalizable to the entire Medicare population. However, this study demonstrated that patients at high risk of hospitalization engaged in CRN behaviors different from those in the general Medicare population. In addition, many national surveys are under-powered to study minority groups with high resource utilization. This study provides information on the African American population, which is often underserved and has increased medical needs. The risk profiles for persistence in CRN developed in this study may provide insights into the general population. In addition, this study had limited power to detect the difference in CRN by socioeconomic strata despite a relatively large sample size.

## Conclusions

In this cohort study, Medicare beneficiaries at high risk of hospitalization had higher rates of CRN than the general Medicare population despite having insurance coverage. A significant proportion of individuals reported persistent CRN, and the number of patients with transient and persistent CRN differed between those younger than 65 years and those 65 or older. More research appears to be needed to understand CRN patterns in this population to formulate health and social policies to identify and target those with persistent CRN and improve the efficiency of interventions.
